# An Evaluation Framework for Large Language Models in Clinical Nursing: A Scoping Review and Expert Consultation

**DOI:** 10.1155/jonm/9524014

**Published:** 2026-09-02

**Authors:** Yingzhuo Ma, Shangqin Liu, Jingyan Song, Xiaobo Song, Xiaoling Yang, Qinghua Zhao, Mingzhao Xiao, Jun Wang

**Affiliations:** ^1^ Department of Nursing, The First Affiliated Hospital of Chongqing Medical University, 1 Youyi Road, Chongqing 400016, China, cqmu.edu.cn

**Keywords:** clinical nursing practice, expert consultation, large language model, scoping review

## Abstract

**Aim:**

To examine the current state of large language model (LLM) evaluation in clinical nursing, synthesize the evaluation dimensions and metrics reported in the literature, and develop a preliminary evaluation framework for LLMs in clinical nursing through expert consultation.

**Background:**

The advancement of artificial intelligence products, exemplified by LLMs, has generated excitement about their potential applications in clinical nursing practice, but their effectiveness remains uncertain.

**Methods:**

A scoping review was conducted in accordance with Arksey and O’Malley’s framework and incorporated experts’ consultation. A literature search was conducted across Web of Science, PubMed, Embase, and the Cochrane Library, from their inception to June 21, 2025. Three expert meetings involving eight experts were conducted between August and October 2025 to synthesize evaluation frameworks and scenarios.

**Results:**

A total of 42 studies were included, and the GPT family was the most frequently evaluated. Thirty‐seven evaluation metrics were extracted and refined through expert consultation into six primary domains: performance and accuracy, clinical validity and safety, workflow integration and efficiency, usability and user experience, model reliability and ethical considerations, and competency development. A scenario classification and a proposed minimum technical reporting checklist were also developed to support the transparent and comparable evaluation of LLMs in clinical nursing.

**Conclusion:**

This study used a scoping review and expert consultation to summarize contemporary literature on LLM evaluations in clinical nursing practice. It provides a preliminary, structured basis for developing and refining a standardized evaluation framework.

**Implications for Nursing Management:**

This study highlights the need for systematic and context‐sensitive evaluation of LLMs in clinical nursing. The findings provide nursing managers with a structured reference for identifying core evaluation dimensions, interpreting evidence, and planning the evaluation and deployment of nursing‐specific LLM applications.

## 1. Introduction

Over the past 4 decades, artificial intelligence (AI) has increasingly been integrated into clinical nursing practice and has demonstrated broad potential [1]. The earliest publications on this topic indexed in MEDLINE date back to 1985, when research primarily focused on expert systems for clinical decision support [2]. Research subsequently expanded from nurse scheduling models to machine learning–based clinical risk prediction and natural language processing [3, 4]. This wave of innovation has pushed intelligent applications in clinical nursing to new heights, with large language models (LLMs) representing one of the most significant advances in the field in recent years [5].

While the basic components of LLMs have long been established, the introduction of ChatGPT in November 2022 marked a crucial shift by greatly increasing public access to LLMs and stimulating considerable interest in their potential applications in clinical nursing [5, 6]. Fundamentally, LLMs represent a novel class of AI based on the transformer architecture. These systems operate as deep neural networks with parameters ranging from billions to trillions, having been pretrained on vast repositories of text data such as digital books, articles, and diverse internet content [7, 8]. Through the training of specialized medical and nursing corpora, LLMs achieve near‐clinical expert performance in various clinical tasks [9]. Previous research highlights the potential role of LLMs as critical adjunctive tools in nursing practice, particularly within underserved regions facing workforce deficits [10, 11]. Consequently, healthcare systems are increasingly incorporating these technologies into comprehensive care models to bridge the gap between limited staffing and escalating patient needs [12].

However, as general‐purpose LLMs are increasingly applied in clinical nursing, concerns about hallucinations have become more prominent. Given the highly specialized and high‐stakes nature of clinical nursing, such errors may hinder the safe and effective integration of LLMs into practice [13]. To address these concerns, multiple complementary strategies have been adopted, including domain‐specific pretraining or fine‐tuning (FT), retrieval‐augmented generation (RAG), knowledge graph‐based grounding, prompt engineering (PE), and human verification [11]. Moreover, a recent study indicated that RAG is increasingly adopted in medical and nursing applications; it enables LLMs to incorporate external domain‐specific knowledge without retraining the underlying model [14]. These approaches may improve the factual grounding, domain relevance, and traceability of model outputs, thereby enhancing their suitability for clinical nursing tasks [11]. At the same time, successive model updates and iterations have improved the performance of LLMs, strengthening their ability to undertake an expanding range of clinical nursing tasks.

With the continued development of optimization strategies and iterative improvements in model performance, research on LLM applications in clinical nursing has gradually expanded to areas such as clinical decision support, patient education, nursing documentation generation, and workflow optimization [11]. Building on these expanding applications, existing evaluation research in healthcare can be broadly characterized in three ways. First, many studies rely primarily on automated or accuracy‐centered metrics, often using medical question‐answering and diagnostic benchmarks, whereas evaluations based on real patient‐care data remain limited [15]. Second, general healthcare evaluation frameworks have been proposed to standardize human evaluation. For example, QUEST covers information quality, understanding and reasoning, expression, safety, and trust across the planning, implementation, and review stages of evaluation [16]. Third, several frameworks have been developed for specific medical tasks. CRAFT‐MD focuses on conversational clinical reasoning, history‐taking, and diagnostic accuracy in patient interaction tasks [17], whereas the framework developed by Seo et al. evaluates the accuracy and clinical applicability of LLM‐generated medical documentation [18]. However, a comprehensive framework for the multidimensional evaluation of LLMs in clinical nursing remains lacking.

Given the urgent need for standardization, this study aims to map existing gaps in evaluation methods, synthesize the evaluation dimensions and metrics reported in the literature, and develop a preliminary evaluation framework for LLMs in clinical nursing through expert consultation.

## 2. Methods

To thoroughly identify the evaluation metrics, situations, and efficacy of LLMs in clinical nursing, we employed a scoping review methodology enhanced by expert consultation. The methodology was underpinned by the framework originally proposed by Arksey and O’Malley [19] and further informed by Levac et al. [20], who emphasize the critical value of the consultation phase. Accordingly, our study progressed through six methodological stages: (1) defining research questions, (2) identifying relevant studies, (3) study selection, (4) charting the data, (5) collating, summarizing, and reporting the results, and (6) conducting expert meetings to refine and contextualize the findings. This study was reported in accordance with the PRISMA‐ScR checklist [21]. The review protocol was publicly registered on the Open Science Framework and is available at https://osf.io/24jvt/metadata/osf.

### 2.1. Identifying the Research Questions

This study aimed to examine the current state of LLM evaluation in clinical nursing, synthesize the evaluation dimensions and metrics reported in the literature, and develop a preliminary evaluation framework for LLMs in clinical nursing through expert consultation. For this review, clinical nursing tasks were defined as specific nursing‐related activities in which LLMs were used to support, assist, or perform functions within clinical practice, clinical nursing management, or patient care. Evaluation was broadly defined as any empirical assessment of a general‐purpose, domain‐adapted, or technically optimized LLM, or an LLM‐based system, including its generated outputs, task performance, technical configuration or optimization strategy, interaction with users, and effects in nursing‐related contexts. The guiding research questions were as follows:

1. What types of LLMs and technical approaches have been evaluated in clinical nursing?
2. In which clinical nursing tasks and contexts have LLMs been evaluated?
3. What evaluation methods, dimensions, and metrics have been reported?
4. What effectiveness and outcomes have been identified in the existing literature?

### 2.2. Identifying Relevant Studies

Search strategies were formulated through a collaborative process involving an academic librarian and two researchers, informed by a preliminary review of the literature. We executed comprehensive searches across PubMed, Embase, the Cochrane Library, and Web of Science, covering records from their inception to June 21, 2025. To mitigate publication bias, we supplemented this with a search of Google Scholar to capture gray literature, such as dissertations, and manually screened bibliographies of retrieved items [22]. Detailed search strategies for each database are provided in Appendix 5. Recognizing that LLM application in clinical nursing is a nascent field with nonstandardized terminology, we adopted a two‐phase search strategy. Initially, a broad query focusing on clinical nursing and LLMs was used to maximize sensitivity. Subsequently, we applied strict eligibility criteria, where two researchers independently screened titles, abstracts, and full‐texts to identify studies specifically evaluating the utility and performance of LLMs in this domain.

### 2.3. Eligibility Criteria for Study Selection

#### 2.3.1. Eligibility Criteria

We established eligibility criteria based on the population, concept, and context (PCC) framework [23]. Studies were eligible if they met the following criteria: (1) Population: the review targeted nursing professionals in clinical settings, including registered nurses, interns, and managers. Technical evaluations of LLMs focused on clinical nursing applications were also included, even without human participants. (2) Concept: articles were selected if they evaluated the efficacy or performance of LLMs and related technologies within clinical nursing domains. (3) Context: There were no geographical or sociocultural restrictions.

Studies were excluded if they (1) included mixed clinical populations but did not report nursing‐specific data separately; (2) were published in languages other than English; (3) were nonempirical in nature, including study protocols, reviews, editorials, comments, or conference abstracts; (4) had no accessible full‐text version; or (5) evaluated LLMs solely using engineering benchmarks, such as parameter size, hardware‐dependent throughput, and memory consumption, without assessing their performance in a specific clinical nursing task or context.

#### 2.3.2. Study Selection

**FIGURE 1:**
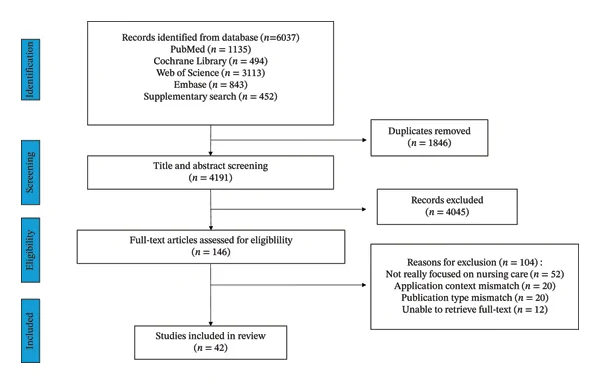
Preferred Reporting Items for Systematic Reviews and Meta‐Analyses 2020 flow diagram.

The studies retrieved were imported into EndNote 21 to ensure the removal of duplicate entries. Subsequently, the first author and co‐first author independently screened the literature in two phases. They first evaluated titles and abstracts for relevance, followed by a full‐text review based on the established eligibility criteria. Any conflicts were adjudicated by a third author to reach a consensus. Reasons for exclusion were documented for each excluded article, with the overall screening process illustrated in Figure 1. This dual screening approach enhances the reliability of study selection and aligns with recognized methodological standards [24].

### 2.4. Charting the Data

Data extraction was performed using a structured Excel form that included the following fields: (1) study characteristics: article title, author(s), source journal, year of publication, country, and study aims, as detailed in Appendix 1; (2) model information, including (2.1) model type; (2.2) specific model name; and (2.3) the definition and architecture of the model as described by the authors of the included study; (3) major scenarios evaluated: classification of the primary clinical task the LLM was evaluated for; (4) evaluation methods or tools: details of how the evaluation was conducted, including the overall approach, the specific metrics used, and any validated tools or scales employed; and (5) clinical effectiveness.

Data extraction was performed by the first reviewer and validated by a second reviewer. Any inconsistencies were settled through discussion to achieve consensus. All figures were created using R software (Version 4.5.1).

### 2.5. Collating, Summarizing, and Reporting the Results

Given the heterogeneity of the included studies, a narrative synthesis approach was adopted. We conducted a descriptive analysis of the study characteristics. For the evaluation metrics and scenarios, we employed content analysis to group diverse indicators into cohesive domains [25]. This step provided the preliminary framework for the subsequent expert consultation.

### 2.6. Consultation

An iterative consultation process comprising three rounds of meetings was conducted between August and October 2025. Initially, the research team synthesized a preliminary list of metrics and scenarios based on the scoping review findings through internal group discussions. In the first round, experts evaluated this preliminary list for comprehensiveness and clarity, during which they added missing indicators, removed irrelevant items, modified wording for clinical precision, and reclassified items into appropriate domains. Following an interim synthesis by the research team to incorporate this feedback, a second round was held to resolve disagreements and further refine the classification of metrics into primary domains. Finally, a third round was conducted to reach a consensus regarding the final structure of the evaluation framework, the specific inclusion of metrics, and the definitions of clinical application scenarios. Consensus was reached through iterative discussion until no substantive disagreements remained.

## 3. Results

Figure 1 illustrates an overview of the review process. The preliminary search produced 6037 items. After a series of repeated screenings, 42 studies were deemed appropriate for extraction and final assessment. To ensure the professionalism and comprehensiveness of the evaluation framework, we organized a total of three expert meetings. Eight experts were recruited from three healthcare institutions and one university. All experts held professional titles at the intermediate level or above, and the panel consisted of 2 males and 6 females. Their professional domains included medical and nursing management (*n* = 2), nursing informatics (*n* = 3), and clinical nursing practice (*n* = 3). Experts were selected based on their professional standing and practical experience in these relevant fields. Through discussion and consensus from the expert meetings, we finalized a scenario classification for evaluating LLMs, which includes 5 primary indicators and 14 secondary indicators (see Appendix 4). Concurrently, a comprehensive evaluation framework comprising 6 primary indicators and 37 secondary indicators was established (see Appendix 3). To complement this evaluation framework and improve the transparency, reproducibility, and comparability of LLM‐based studies in clinical nursing, a minimum technical reporting checklist was also developed. The checklist recommends reporting, where applicable, the model name and version, access mode, prompting strategy, parameter settings, input data types, technical optimization methods, knowledge sources, output formats, and reproducibility‐related information (see Appendix 6).

### 3.1. Study Characteristics

The characteristics of the included studies are presented in Appendix 1. The articles mentioned were published between 2023 and 2025. Of them, 42 were peer‐reviewed articles, comprising cross‐sectional studies (13), descriptive studies (8), experimental studies (7), mixed‐methods studies (3), observational studies (2), a qualitative study (1), a debate essay (1), a retrospective cohort study (1), and others (6). Regarding the country of origin, China contributed 13 studies, followed by the United States (6) and Korea (4). There were three studies each from Australia, Israel, and Turkey; two each from Italy, Spain, and Japan; and one each from Slovenia, the United Kingdom, Germany, and Iran, as illustrated in Appendix 1.

### 3.2. Model Information

In the section concerning model information, we first analyzed the types of LLMs evaluated, the technical optimization strategies employed, whether the studies involved system development or application, and the final output modalities, as illustrated in Appendix 2.

**FIGURE 2:**
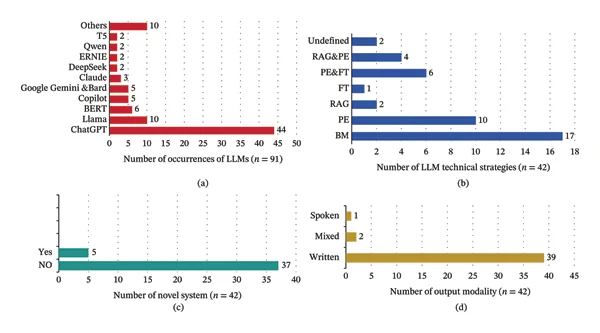
Statistical analysis of LLMs. Note: The KG‐based approaches identified in the studies included in this review incorporated knowledge graphs as external retrieval and grounding layers within RAG pipelines. Therefore, they were classified as KG‐enhanced RAG within the broader RAG category. However, this operational classification does not imply that all KG‐based approaches are equivalent to or should be subsumed under RAG, as standalone KG reasoning, KG‐guided inference, and neuro‐symbolic architectures represent conceptually distinct approaches.

The studies evaluated a total of 91 instances of LLMs. Among these, models from the GPT family were the most frequently evaluated, accounting for 44 records (48.35%). Llama was assessed in 10 records (10.99%). Other models are illustrated in Appendix 2 and Figure 2a. Some researchers adopted various technical strategies to enhance their performance in professional clinical environments. We categorized these strategies into three types: (1) Base model (BM) group: a total of 17 studies (40.47%) directly applied base models without specific optimization to evaluate the performance and limitations of general‐purpose LLMs in clinical nursing practice. These studies found that they exhibit significant deficiencies in factual accuracy, depth of domain knowledge, and adherence to clinical nursing guidelines [26–42]. (2) Single‐technique optimization (13, 30.95%), where single‐technique refers to the use of one of PE, RAG, or model FT. PE: 10 articles utilized PE to constrain the model’s output format and guide its reasoning process, thereby improving the controllability and conformity of the generated content [43–52]. RAG: 2 studies employed RAG to address the model’s knowledge limitations and information hallucination issues [53, 54]. By dynamically connecting the model with credible knowledge sources, such as clinical guidelines and recent research literature, this approach provides factual grounding for the model’s responses, significantly enhancing its accuracy and reliability. FT: one study adopted FT strategies; by training models on specialized nursing corpora, FT enables the model to internalize domain‐specific terminology, communication styles, and tacit knowledge. (3) Composite technique optimization (10, 23.81%), defined as strategies that integrate two or more optimization techniques. Furthermore, 10 studies implemented composite strategies to achieve synergistic effects. Four of these combined RAG with PE to ensure both information accuracy and standardized output formats [55–58]. The other six studies explored the combination of FT and PE [59–64]. Finally, two articles did not specify their technical optimization strategy [65, 66], as illustrated in Appendix 2 and Figure 2b.

Furthermore, we reviewed existing LLM‐based platforms and systems in clinical nursing practice. Most research centered on applying or optimizing general‐purpose LLMs (37, 88.10%), as opposed to creating novel platforms and systems (5, 11.90%). Two studies focused on the nursing documentation management system, which was designed to generate nursing records or provide nursing diagnosis recommendations for improved efficiency [65, 66]. Others included obstetric nursing communication simulation training [59], outpatient reception and triaging [64], and optimizing clinical workflows, such as specimen submission [67], as illustrated in Appendix 2 and Figure 2c.

Correspondingly, in terms of output modality and interaction, the predominant approach remains text‐based output (39, 92.86%). Only one study (2.38%) featured a model designed for multiturn, interactive dialog with users. Additionally, two studies (4.76%) explored multimodal integration, incorporating visual and auditory elements to provide a more information‐rich and versatile assistive interface, as illustrated in Appendix 2 and Figure 2d.

### 3.3. Evaluation Methods

**FIGURE 3:**
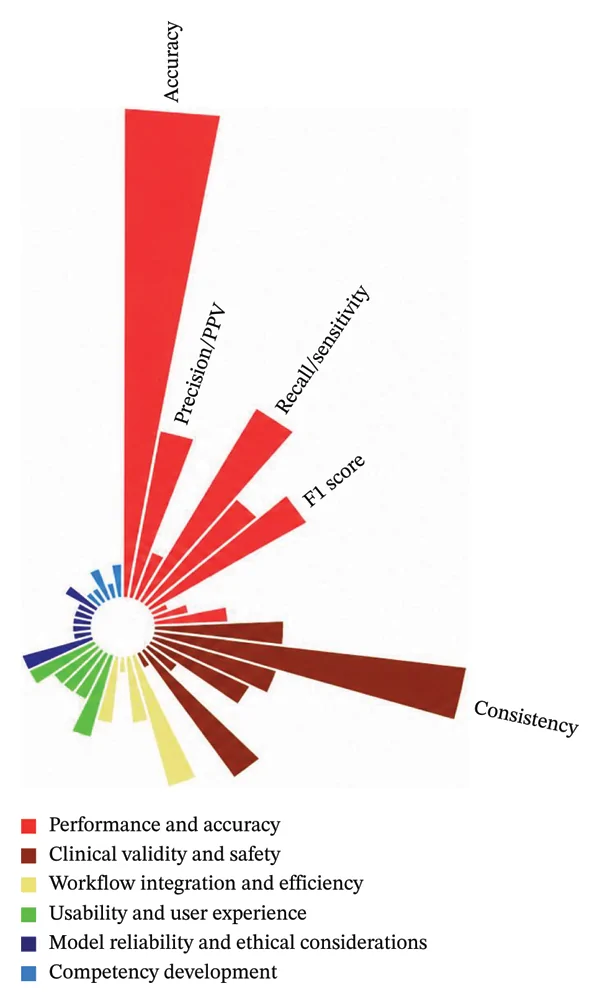
Distribution and categorization of evaluation metrics.

Assessing LLMs in practical nursing practice requires a thorough and varied approach that captures the intricacies of the nursing specialty. Researchers have utilized diverse techniques, frequently incorporating both quantitative and qualitative indicators, to comprehensively assess the performance of LLMs. This section examines all tactics and considerations identified in the analyzed studies. Through a series of expert panel meetings, we categorized the 37 evaluation metrics extracted from the literature into six primary domains: performance and accuracy, clinical validity and safety, workflow integration and efficiency, usability and user experience, model reliability and ethical considerations, and competency development. A comprehensive analysis of evaluation parameters across the 42 studies, as summarized in Appendix 3 and Figure 3, revealed that “performance and accuracy” and “clinical validity and safety” were the most frequently evaluated dimensions. Among the 42 articles reviewed, five specific metrics were mentioned 10 or more times: accuracy (*n* = 29, 69.05%), consistency (*n* = 17, 40.48%), recall/sensitivity (*n* = 13, 30.95%), precision/PPV (*n* = 10, 23.81%), and *F*1 score (*n* = 10, 23.81%).

The analysis logically begins with the foundational dimension of “performance and accuracy,” which quantitatively measures a model’s effectiveness on clinical nursing tasks against a definitive “gold standard.” This domain is primarily concerned with objective metrics in classification, prediction, and information retrieval tasks, including accuracy, precision/PPV, NPV, recall/sensitivity, specificity, *F*1 score, *F*2 score, BERT score, and AUC. However, high quantitative performance alone does not guarantee clinical utility. Therefore, the second dimension, “clinical validity and safety,” is crucial. It assesses the clinical appropriateness, quality, and potential for harm in the model’s generated content. This evaluation is typically qualitative and requires subjective assessment by clinical experts to ensure the output is sound, safe, and contextually relevant to patient care, encompassing metrics such as factuality, consistency, completeness, relevance, clinical prioritization, error type, and guidance. Beyond being clinically sound, an LLM must be practical for daily use. This leads to the dimension of “workflow integration and efficiency,” which evaluates the model’s impact on existing nursing workflows. This domain measures the degree to which the LLM streamlines processes, reduces administrative burden, and enhances productivity in a real‐world setting, using indicators such as response time, task completion efficiency, communication efficiency. The success of workflow integration is directly tied to how the tool is perceived by its users. Consequently, the “usability and user experience” dimension assesses the subjective experience of clinical end‐users. This involves gauging their satisfaction, perceived usefulness, and ease of use, as well as the affective interaction quality and fluency of the model. Underpinning all these user‐facing aspects is the fundamental need for trustworthiness. The “model reliability and ethical considerations” dimension addresses the soundness and dependability of the LLM, examining its output stability, robustness, fairness/bias, and explainability. It also scrutinizes for potential issues such as speculative vocabulary, variable performance based on seniority, and inconsistent accuracy across different time periods. Finally, considering the long‐term impact, the “competency development” dimension evaluates how LLMs influence the professional growth of clinical users. This user‐centric domain focuses on enhancing clinical knowledge, skills, and overall competence, including AI literacy, usage rate, and improvements in communication skills and critical thinking, thereby assessing the educational and developmental value of the tool itself. For specific literature references and concept citations, please refer to Appendix 3.

### 3.4. Major Scenarios Evaluated

**FIGURE 4:**
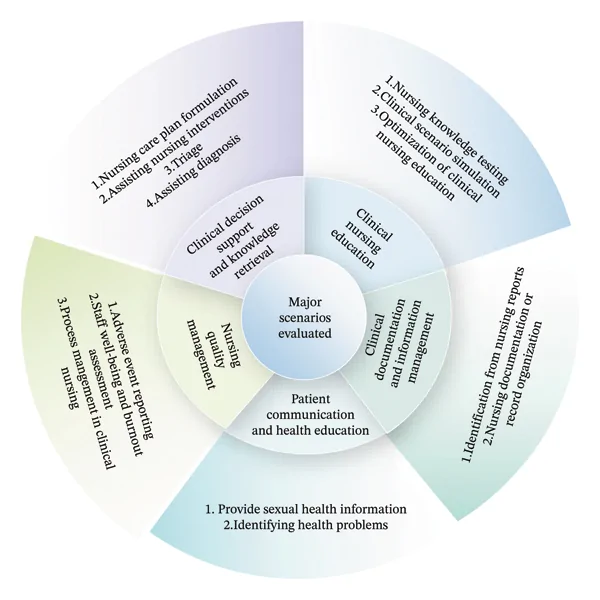
Major scenarios evaluated in clinical nursing.

Existing scenario evaluation of LLMs in clinical nursing can be categorized into five main types based on their functional roles: (1) clinical documentation and information management (*n* = 8, 19.05%), (2) clinical decision support and knowledge retrieval (*n* = 18, 42.86%), (3) patient communication and health education (*n* = 2, 4.76%), (4) clinical nursing education (*n* = 11, 26.19%), and (5) nursing quality management (*n* = 3, 7.14%). Detailed classifications are presented in Appendix 4 and Figure 4.

Among these, evaluation in clinical documentation and information management [37, 48, 50, 56, 57, 61, 62, 66] encompasses automated generation of nursing records, pathology and clinical reports, and information extraction and structuring. In clinical decision support and knowledge retrieval [26, 31–34, 38, 41, 43–47, 51–54, 64, 65], LLMs are utilized for question answering, assisting in the formulation and implementation of nursing care plans, and supporting differential diagnosis and symptom analysis. Patient communication and health education [49, 55] applications primarily focus on enhancing nurse–patient interactions and delivering personalized health education. In clinical nursing education [27–30, 35, 36, 39, 40, 42, 58, 59], LLMs have been applied in nursing knowledge assessment, simulation‐based clinical training, and personalized learning. In nursing quality management [60, 63, 68], LLMs can be used for computationally detecting fall events to ensure patient safety. Furthermore, LLMs can discern theoretically based burnout symptoms from nurses’ remarks in extensive surveys and examine their correlations with vaccine hesitancy and health beliefs, thus supporting nurses’ well‐being and the consistency of nursing quality.

### 3.5. Clinical Effectiveness

The reviewed literature demonstrates a diverse range of evaluations of LLMs across key domains of clinical nursing practice. Given the heterogeneity in study designs, evaluation metrics, and clinical contexts, direct comparisons of performance remain challenging. Therefore, this section provides an integrative summary of current evidence regarding LLM applications in clinical documentation and information management, clinical decision support and knowledge retrieval, patient communication and health education, clinical nursing education, and nursing quality management.

In the domain of nursing documentation, multiple studies have demonstrated that LLMs can effectively automate and enhance record generation, quality control, and information retrieval [48, 65, 66]. Their implementation has led to substantial reductions in documentation time, omission rates, and inconsistency errors. Moreover, LLM‐based systems were able to accurately extract and summarize clinical details from unstructured notes, improving the completeness and readability of nursing records. Although further optimization is needed for nuanced or context‐specific data, these findings support the feasibility of LLMs as assistive tools for clinical documentation management.

Recent studies have explored the application of LLMs in clinical triage and patient prioritization. Evidence indicates that models such as ChatGPT and Copilot achieved diagnostic and classification accuracy comparable to or slightly below that of experienced triage nurses [41, 51, 54]. These tools demonstrated particular strengths in identifying high‐acuity patients and optimizing patient flow, although they occasionally tended to overtriage. Advanced versions such as GPT‐4‐Turbo exhibited near‐perfect accuracy through improved prompting strategies. Collectively, these findings suggest that integrating LLMs into triage processes can enhance efficiency and reliability, serving as a valuable decision‐support mechanism when combined with clinical judgment.

Recent research has highlighted the growing role of LLMs in supporting patient communication and health education. Conversational AI systems have shown potential to improve patients’ understanding of health information, enhance engagement, and promote adherence to treatment plans. Studies evaluating AI chatbots for health counseling reported that these tools were able to deliver medically accurate, empathetic, and accessible information, particularly for sensitive topics such as sexual and reproductive health [55]. However, researchers cautioned that AI‐assisted communication should complement, rather than replace, professional consultation, as LLMs may occasionally provide incomplete or contextually inappropriate advice [69]. Overall, evidence suggests that integrating LLM‐based chatbots and education tools into healthcare communication workflows can enhance patient empowerment, foster trust, and improve the quality of health education delivery.

LLMs have also been widely adopted in clinical nursing education, particularly in simulation‐based learning and blended teaching models. Intervention studies have shown that the inclusion of LLM tools significantly improved nursing students’ AI literacy, report writing, and critical thinking skills [36, 40, 59]. Virtual reality–based learning environments driven by conversational AI enhanced communication confidence and engagement, while blended teaching models supported deeper clinical reasoning and reflective learning. Overall, these results highlight the potential of AI‐assisted educational models to foster more active and competency‐based nursing education.

LLMs have also contributed to nursing quality management and patient safety initiatives. Their deployment in monitoring systems and event reporting has led to measurable improvements in data integrity and error detection [60, 63, 68]. AI‐enhanced platforms reduced omission rates in nursing documentation, increased the accuracy of adverse event reports, and provided data‐driven insights for workforce well‐being, such as the detection of burnout symptoms among healthcare professionals [63]. These applications highlight the growing role of LLMs in supporting evidence‐based quality improvement and promoting safer clinical practice environments.

## 4. Discussion

This review aimed to synthesize the existing literature on current and potential evaluation of LLMs across clinical nursing tasks. To achieve this, the included literature was systematically organized and analyzed according to four key domains: model information, major scenarios evaluated, evaluation methods, and clinical effectiveness.

In the section concerning model information, we first analyzed the types of LLMs evaluated, the technical optimization strategies employed, whether the studies involved system development or application, and the final output modalities. Our findings indicate that most studies were dedicated to evaluating the performance of unoptimized GPT‐series models on clinical nursing tasks, which is consistent with findings of Shool et al. [70]. However, these studies almost unanimously concluded that general‐purpose models exhibit deficiencies in factual accuracy, depth of nursing knowledge, and adherence to clinical practice guidelines, a consensus that aligns with the broader existing literature [71, 72]. This limitation has led researchers to adopt model optimization strategies (PE, RAG, and model FT) to address issues such as domain knowledge deficits and hallucinations, which supplements the work of Hobensack et al. [5]. However, our review revealed that existing research demonstrates a clear predilection for “lightweight” optimization. PE is the predominant strategy, which is a method for constraining and guiding model output, not for fundamentally enhancing its core capabilities [73]. In contrast, RAG and model FT techniques that can genuinely address knowledge limitations and hallucinations remain severely underutilized [74]. In addition to RAG and model FT, recent research indicates that integrating LLMs with knowledge graphs is becoming an increasingly common approach in healthcare AI [75]. By combining the generative capabilities of LLMs with structured representations of clinical knowledge and relationships, this approach may improve knowledge grounding, evidence traceability, and multistep reasoning. These capabilities are particularly relevant to clinical nursing, where model outputs should align with nursing processes, clinical practice guidelines, and standardized nursing terminologies [75]. This reliance on low‐cost PE, coupled with the insufficient exploration of techniques like RAG and model FT, may impede the advancement of LLMs toward greater reliability and professionalization in clinical nursing [76]. Therefore, future research should move beyond prompt‐only optimization and further investigate RAG, model FT, and knowledge graph–enhanced systems.

The results indicate that assessments of LLMs in clinical nursing practice often lack rigorous clinical validation and standardized outcome measures. This finding is consistent with Hua et al. [6], who reported that evidence on the evaluation of LLMs in mental health care remains limited and heterogeneous across studies. These limitations hamper the ability to draw definitive conclusions, while the inconsistent use and interpretation of measurement methods further complicate the evaluation of LLMs [77]. To address these challenges, we developed a six‐domain evaluation framework through expert consultation. This framework aligns with the emerging consensus that the evaluation of LLMs in clinical nursing should be multifaceted and practice‐centric [11]. Our findings indicate that most current studies focus primarily on performance and accuracy. This emphasis on accuracy is consistent with a common characteristic of the early‐stage development of medical AI [78, 79]. However, as many scholars have noted, high accuracy does not directly equate to clinical utility [80]. Clinical validity and safety was the second most evaluated dimension. Researchers incorporated subjective assessments by clinical experts to judge the factuality, relevance, and potential risks of model outputs. This indicates a shift in evaluation from “is it technically correct?” to “is it clinically appropriate?” Moreover, a key finding of this review is that the dimensions determining whether a technology can be clinically implemented remain underassessed. For instance, the evaluation of “workflow integration and efficiency” and “usability and user experience” dimensions is notably insufficient, which contrasts with principles emphasized in implementation science and human–computer interaction [81, 82]. Previous research has shown that, for frontline nurses, ease of use, response time, and integration into clinical workflows are as important as algorithmic performance [83, 84]. Likewise, clinical trust will be difficult to build if the long‐term effects of LLM use on clinical judgment and the fairness and transparency of model decisions remain unclear [85]. Future evaluation frameworks for clinical nursing should more explicitly reflect the distinctive nature of nursing practice. Specifically, such frameworks should assess whether LLMs can follow the nursing process, prioritize nursing actions according to patient risk, and generate structured outputs aligned with nursing documentation standards and standardized or local nursing terminologies.

Current evidence indicates that LLM evaluation in clinical nursing spans a broad range of scenarios and dimensions, including documentation, decision support, education, patient communication, and quality management. Among these domains, clinical decision support and knowledge retrieval (*n* = 18, 42.86%) have been most extensively evaluated. In contrast, areas such as patient communication and nursing quality management remain underexplored, which may partly reflect challenges in accessing real‐world patient data, ensuring patient safety, and measuring subjective outcomes. Few studies evaluated LLMs using real‐world clinical data or within live nursing workflows. Aydin et al. [69] similarly found that the literature was concentrated on generating patient education materials and answering patient questions, whereas studies addressing doctor–patient interaction accounted for only 2.5% of the included articles. More broadly, Artsi et al. [86] identified only four empirical studies in which LLM‐enabled tools had been integrated into real‐world clinical workflows, highlighting the limited state of clinical implementation. Future research should therefore prioritize the real‐world validation of LLM‐based tools. The need for real‐world validation also highlights several nursing informatics requirements that may be overlooked in isolated experimental evaluations. When LLMs are integrated into clinical nursing workflows, evaluation should extend beyond task‐level performance to consider information completeness and provenance, the quality and standardization of structured outputs, auditability, interoperability with electronic health records and nursing information systems, and the effects of LLM use on documentation burden [87, 88]. These properties are related to clinical validity and safety, model reliability and ethical considerations, and workflow integration and efficiency and should be more explicitly operationalized in future refinements of the framework.

By classifying research scenarios, this study analyzed the evaluation effectiveness across different contexts. These findings indicate a core trend: LLMs are transitioning from conceptual technologies into practical assistive tools that can empower clinical nursing and improve quality and efficiency. Their value is primarily demonstrated in optimizing workflows, enhancing decision‐making accuracy, and innovating educational models [89]. This aligns with widespread calls to reduce administrative burdens and alleviate burnout, potentially enabling nurses to reinvest their efforts into core tasks demanding professional judgment and humanistic care [90]. However, while existing research collectively highlights the promise of LLMs in clinical nursing, the pervasive heterogeneity in study designs and evaluation metrics makes direct cross‐study performance comparisons extremely difficult. This challenge is not uncommon in rapidly emerging technology fields, but it severely impedes the systematic synthesis of evidence and the pace of clinical translation [5]. Furthermore, this heterogeneity is exacerbated by methodological flaws in some studies, particularly those conducted via public LLM web interfaces, leading to insufficient reproducibility and generalizability of the findings [39]. Therefore, future research needs to focus on establishing standardized evaluation frameworks and uniform performance metrics tailored to nursing‐specific scenarios. This is crucial for scientifically assessing the effectiveness, safety, and fairness of different models in specific clinical contexts. Moreover, research should move beyond cross‐sectional accuracy assessments; more high‐quality longitudinal and real‐world studies are urgently needed to evaluate the long‐term impacts of LLM integration into clinical work, including the comprehensive effects on patient health outcomes, nurse job satisfaction, human–computer interaction dynamics, and healthcare costs.

Future research should proceed in two complementary directions. On the one hand, greater attention should be given to developing specialized agent‐based systems capable of decomposing complex tasks, coordinating multistep workflows, invoking external tools, and retrieving information from clinical knowledge sources. These systems may extend LLM applications beyond isolated question answering toward more comprehensive clinical nursing support, while conversational and multimodal interfaces may facilitate their use in real‐world nursing settings. Their evaluation should consider not only task performance but also agent coordination, degree of autonomy, and compatibility with clinical workflows. On the other hand, future research should examine how evaluation findings can be translated into governance processes embedded within clinical nursing workflows. This includes clarifying responsibilities for reviewing and validating model outputs, establishing escalation pathways for high‐risk or uncertain recommendations, and developing mechanisms for documenting, tracing, and learning from AI‐related errors. Hospital administrators and nursing managers should coordinate these processes and define the respective responsibilities of clinical nurses, physicians, technical personnel, and legal or ethics professionals. When AI‐generated information is used in patient education or communication, patients and families should also be appropriately informed.

## 5. Limitations

This study has several limitations. First, given the rapid expansion of research on LLMs, relevant studies published after June 2025 or not identified through our search strategy may have been missed. In addition, global policies, professional guidance, and clinical perspectives concerning LLMs continue to evolve, which may affect the continued applicability of some findings. Second, the proposed framework was constrained by the scope and quality of the available evidence. Knowledge representation specific to clinical nursing practice was insufficiently and inconsistently addressed in the included studies. Although some related elements may be reflected within clinical validity and safety, the available evidence did not support its clear definition as a distinct dimension or subdimension. Further evidence synthesis and expert consultation are needed to clarify this construct and determine how it should be incorporated into future revisions of the framework. Third, although the expert panel included participants from three healthcare institutions and one university and represented several relevant professional domains, its limited geographical coverage may have introduced selection bias and restricted the broader representativeness of the findings. Finally, the available evidence was insufficient to establish validated scenario‐specific indicators, priorities, or weights. Therefore, the proposed framework and minimum technical reporting checklist should be regarded as preliminary tools rather than formal evaluation guidelines. Larger, multicenter, and geographically diverse studies are needed to further refine and prospectively validate these tools across different clinical scenarios, institutions, LLM applications, and user populations.

## 6. Conclusions

This study synthesized contemporary evidence and expert perspectives to propose a preliminary framework for the evaluation of LLMs in clinical nursing practice. The findings demonstrate the transformative potential of LLMs while identifying persistent challenges related to ethical risks, inconsistent evaluation approaches, and the limited representation of nursing‐specific requirements. The proposed framework and minimum technical reporting checklist provide a structured basis for evaluating the safety, effectiveness, and equity of LLM applications in clinical nursing. Further refinement and prospective validation are required before these tools can be adopted as formal evaluation guidelines.

## Author Contributions

Yingzhuo Ma: writing–review and editing, writing–original draft, project administration, methodology, investigation, formal analysis, data curation, and conceptualization. Shangqin Liu: writing–review and editing, writing–original draft, formal analysis, data curation, and conceptualization. Jingyan Song and Xiaobo Song: formal analysis, project administration, and data curation. Xiaoling Yang: formal analysis and data curation. Qinghua Zhao: methodology, supervision, and project administration. Mingzhao Xiao: writing–review and editing, supervision, formal analysis, and conceptualization. Jun Wang: writing–review and editing, supervision, resources, methodology, funding acquisition, conceptualization, and project administration.

## Funding

This work was supported by the 2025 Graduate Education Reform Project of the First Clinical College, Chongqing Medical University (jgxm‐202502), 2024 University‐Level Key Project of Educational and Teaching Reform Research, Chongqing Medical University (JY20240202), and 2024 College‐Level Graduate Supervisor Team Project of the First Clinical College, Chongqing Medical University (CYYY‐DSTDXM‐202409).

## Ethics Statement

The Research Ethics Committee of the first author’s affiliated hospital approved the study (approval number: ZZ2025‐320‐01).

## Conflicts of Interest

The authors declare no conflicts of interest.

## Supporting Information

Additional supporting information can be found online in the Supporting Information section.

## Supporting information


**Supporting Information 1** Appendix 1: Characteristics of the included studies, including publication information, country, and study aims.

**Supporting Information 2** Appendix 2: Model information, including model type, application method, novel systems/applications, and output modalities.

**Supporting Information 3** Appendix 3: The proposed evaluation framework, including evaluation principle, dimension, definition.

**Supporting Information 4** Appendix 4: Evaluation scenarios for LLMs, including Level 1 evaluation scenario and Level 2 evaluation scenario.

**Supporting Information 5** Appendix 5: Detailed search query used for PubMed, Web of Science, the Cochrane Library, and Embase.

**Supporting Information 6** Appendix 6: The proposed minimum technical reporting checklist for studies evaluating LLMs in clinical nursing practice.

**Supporting Information 7** PRISMA_checklist.

## Data Availability

All data generated or analyzed during this study are included in this published article.
