# Supplementary material for: An Evaluation Framework for Large Language Models in Clinical Nursing: A Scoping Review and Expert Consultation
Source: J Nurs Manag. 2026 Sep 2;2026:9524014. doi: 10.1155/jonm/9524014 (PMC13538949; doi:10.1155/jonm/9524014)
Supplement: Supplementary file 1 [file JONM-2026-9524014-s001.docx]

Appendix1. Characteristics of Included Studies

|  | **Author** | **Publication Date** | **journal name** | **Country** | **Article type** | **study aim** |
| --- | --- | --- | --- | --- | --- | --- |
| 1 | (Woodnutt et al. 2024) | 2023-8 | Journal of Psychiatric and Mental Health Nursing | The UK | debate essay | To evaluate and compare a care plan generated by ChatGPT against one created by nurses. |
| 2 | (Wang et al. 2024) | 2024-1 | International Journal of Nursing Studies Advances | China | cross-sectional study | To test if a large language model could effectively summarize unstructured nursing records about cancer wound care and to evaluate the quality of those summaries |
| 3 | (Wu et al. 2024) | 2024-10 | JMIR Medical Education | China | cross-sectional study | To evaluate the performance of large language models in answering NCLEX-RN and NNLE multiple-choice questions across different language inputs |
| 4 | (Gosak et al. 2025) | 2024-11 | journal of nursing scholarship | Slovenia | descriptive study | To evaluate large language models, including ChatGPT-3.5 , Bing Chat , and Google Bard , for generating PICOT clinical questions and developing search strategies |
| 5 | (Xu et al. 2024) | 2024-12 | Scientific Reports | China | experimental study | To develop a breast cancer knowledge base and evaluate retrieval-augmented generation in large language model–based nursing systems. |
| 6 | (Zaboli et al. 2024) | 2024-2 | The American Journal of Emergency Medicine | Italy | descriptive study | To assess the reliability and safety of ChatGPT in the Manchester Triage System compared with healthcare professionals. |
| 7 | (Scroggins et al. 2025) | 2024-7 | journal of nursing scholarship | America | others | To generate synthetic patient–nurse communication with GPT-4 and assess its impact on machine learning performance in identifying health problems. |
| 8 | (Wan et al. 2024) | 2024-7 | Nature Medicine | China | experimental study | To evaluate the feasibility of integrating large language models into outpatient reception through a site-specific chatbot and a nurse–SSPEC collaboration model |
| 9 | (Levin, Suliman, et al. 2025) | 2024-8 | Journal of Clinical Nursing | Israel | mixed‐methods study | To compare the diagnostic accuracy and response patterns of ICU nurses and various generative AI models, and to qualitatively interpret the quantitative findings |
| 10 | (Johnson et al. 2025) | 2025-1 | Journal of Perinatal & Neonatal Nursing | America | descriptive study | To build and demonstrate our ChatGPT-4 prompt to support nurse care planning |
| 11 | (Cho et al. 2025) | 2025-2 | journal of advanced nursing | Korea | observational study | To develop a clinical language model–based method for detecting patient fall events and supplementing existing self-reporting systems |
| 12 | (Zhao et al. 2025) | 2025-3 | Nurse Education in Practice | China | cross-sectional study | To develop a custom GPT tailored for the Chinese Nursing Licensing Exam and evaluate its accuracy and response quality |
| 13 | (Saad et al. 2025) | 2025-3 | Journal of Primary Care And Community Health | Israel | cross-sectional study | To compare the diagnostic accuracy and clinical decision-making of experienced community nurses and advanced generative AI systems in simulated patient scenarios |
| 14 | (Levin, Orkaby, et al. 2025) | 2025-3 | pediatric research | Israel | cross-sectional study | To evaluate the potential of large language models in reducing pediatric medication dosage calculation errors compared with experienced nurses |
| 15 | (Karaçay et al. 2025) | 2025-3 | journal of advanced nursing | Turkey | cross-sectional study | To evaluate the performance of Visual ChatGPT in staging pressure injuries using real patient images |
| 16 | (García-Rudolph et al. 2025) | 2025-3 | Nursing education perspectives | Spain | cross-sectional study | To evaluate two free chatbots and one commercial model using questions from a simulated nursing licensure exam |
| 17 | (Arslan et al. 2025) | 2025-3 | The American Journal of Emergency Medicine | Turkey | observational study | To evaluate the performance of ChatGPT and Copilot in triage within a high-volume urban hospital |
| 18 | (Shah-Mohammadi and Finkelstein 2025) | 2025-4 | Studies in Health Technology And Informatics | America | cross-sectional study | To evaluate the ability of GPT-4, specifically GPT-4o, to extract symptoms and signs from nursing notes in the dataset |
| 19 | (Ju et al. 2025) | 2025-4 | Healthcare Informatics Research | Korea | experimental study | To compare traditional nursing documentation with a generative AI system and assess its impact on efficiency, accuracy, and quality |
| 20 | (Zhu et al. 2025) | 2025-4 | JMIR Medical Informatics | China | cross-sectional study | To evaluate seven large language models on the Chinese Nursing Licensure Examination and assess whether combining their outputs improves accuracy |
| 21 | (Tseng, Huang, and Chen 2025) | 2025-4 | Nurse Education Today | China | experimental study | To evaluate the effectiveness of AI literacy training and the use of ChatGPT and Copilot in academic nursing report writing. |
| 22 | (Soddu et al. 2025) | 2025-4 | nursing reports | Italy | cross-sectional study | To evaluate the accuracy, completeness, and safety of ChatGPT-4 responses on pressure injuries in infants |
| 23 | (Cao et al. 2025) | 2025-4 | BMC Nursing | China | others | To assess the quality of nursing diagnoses and plans generated by a prompt framework across large language models and evaluate their potential clinical applicability |
| 24 | (Liu et al. 2025) | 2025-4 | Journal of the American Medical Informatics Association | America | others | To develop and evaluate a method using large language models and a knowledge graph to triage patient messages requiring emergency care |
| 25 | (Graf, Ritzi, and Schoeler 2025) | 2025-5 | Studies in Health Technology And Informatics | Germany | others | To evaluate the use of large language models for classifying nursing reports |
| 26 | (Chen and Liou 2025) | 2025-5 | Nurse Education in Practice | China | mixed‐methods study | To develop the ChatGPT-based Virtual Reality Obstetric Nursing Communication Simulation System and assess its impact on students’ communication skills and learning experience |
| 27 | (Gilart et al. 2025) | 2025-5 | Nursing Reports | Spain | descriptive study | To compare the quality and efficiency of care plans produced by nursing professionals and an artificial intelligence model |
| 28 | (Latt et al. 2025) | 2025-5 | BMC Public Health | Australia | cross-sectional study | To evaluate the performance of three AI chatbots compared with clinically trained staff in responding to sexual health inquiries |
| 29 | (Kuribara, Hirayama, and Hirata 2025) | 2025-5 | Digit Health | Japan | cross-sectional study | To evaluate the accuracy of LLMs on basic nursing knowledge and identify trends in incorrect answers |
| 30 | (Ghaffari et al. 2025) | 2025-5 | Archives of Academic Emergency Medicine | Iran | qualitative study | To evaluate ChatGPT’s ability to generate realistic simulation scenarios to support faculty in medical education |
| 31 | (Wang et al. 2025) | 2025-5 | Journal Of Medical Internet Research | China | experimental study | To evaluate GPT-4-based ChatGPT models in patient triage using the Modified Early Warning Score and assess GPT-4o’s accuracy in simulated outpatient guidance |
| 32 | (Niyirora et al. 2025) | 2025-6 | JAMIA Open | America | descriptive study | To compare the performance of machine learning models and human experts in mapping unstructured nursing notes to the Nursing Interventions Classification system |
| 33 | (Lv et al. 2025) | 2025-7 | Journal of The American Medical Informatics Association | China | experimental study | To develop a DeepSeek-based system for nursing documentation quality control and evaluate its clinical efficacy |
| 34 | (Omranian et al. 2025) | 2025-7 | Jmir Nursing | America | retrospective cohort study | To use natural language processing to identify burnout symptoms from nurse survey comments and assess their associations with vaccine hesitancy and health beliefs |
| 35 | (Wakabayashi and Seto 2025) | 2025-8 | Studies in Health Technology And Informatics | Japan | cross-sectional study | To explore the potential of nursing records in enhancing pharmaceutical safety by analyzing adverse effects using ChatGPT-4 for data categorization |
| 36 | (Vithanage et al. 2025) | 2025-8 | Computers in Biology And Medicine | Australia | others | To develop a framework to evaluate large language models on nursing notes and compare with retrieval-augmented generation configurations to identify optimal strategies |
| 37 | (Tanrıverdi and Söylemez 2025) | 2025-8 | Nurse Education in Practice | Turkey | descriptive study | To compare student-generated nursing care plans with ChatGPT-generated plans for patients undergoing laparoscopic cholecystectomy |
| 38 | (Y. Kim, Kim, and Choi 2025) | 2025-8 | Studies in Health Technology And Informatics | Korea | descriptive study | To summarize unstructured nursing records on cancer wound management using a large language model and evaluate the quality of the summaries |
| 39 | (Zhang et al. 2025) | 2025-8 | Studies in Health Technology And Informatics | Australia | others | To identify symptoms and triggers of agitation in dementia from nursing notes in residential aged care facilities |
| 40 | (Yin et al. 2025) | 2025-9 | Nurse Education in Practice | China | experimental study | To evaluate the effect of a ChatGPT-driven blended teaching model in nursing rounds |
| 41 | (Wan et al. 2025) | 2025-9 | Disability And Rehabilitation-assistive Technology | China | descriptive study | To optimize specimen submission and reduce errors using predictive analytics, training, and automated feedback |
| 42 | (J. Kim, Won, and Lee 2025) | 2025-9 | Nurse Education in Practice | Korea | mixed‐methods study | To evaluate the use of a GPT-based virtual patient in nursing education |
