# Supplementary material for: An Evaluation Framework for Large Language Models in Clinical Nursing: A Scoping Review and Expert Consultation
Source: J Nurs Manag. 2026 Sep 2;2026:9524014. doi: 10.1155/jonm/9524014 (PMC13538949; doi:10.1155/jonm/9524014)
Supplement: Supplementary file 2 [file JONM-2026-9524014-s002.docx]

**Appendix** 2. Model Information

| Number | Reference | Model type | LLM Application Method | Novel System/Application | Output modality |
| --- | --- | --- | --- | --- | --- |
| 1 | (Woodnutt et al. 2024) | ChatGPT* | Base Model | NO | Written |
| 2 | (Wang et al. 2024) | ChatGPT-3.5、ChatGPT-4 | Prompt Engineering | NO | Written |
| 3 | (Wu et al. 2024) | ChatGPT 3.5, ChatGPT 4, Google Bard* | Base Model | NO | Written |
| 4 | (Gosak et al. 2025) | ChatGPT-3.5, Bing Chat, Google Bard* | Prompt Engineering | NO | Written |
| 5 | (Xu et al. 2024) | ChatGPT-4 | RAG | NO | Written |
| 6 | (Zaboli et al. 2024) | ChatGPT-3.5 | Base Model | NO | Written |
| 7 | (Scroggins et al. 2025) | ChatGPT-4 | Prompt Engineering | NO | Written |
| 8 | (Wan et al. 2024) | ChatGPT3.5 | Prompt Engineering、Model Fine-tuning | SSPEC | Spoken |
| 9 | (Levin, Suliman, et al. 2025) | ChatGPT-4、Claude-2.0 | Base Model | NO | Written |
| 10 | (Johnson et al. 2025) | ChatGPT-4 | Prompt Engineering | NO | Written |
| 11 | (Cho et al. 2025) | ChatGPT-4, BERTBase, mBERT, KLUE-BERT, Med-BERT, Bio+ClinicalBERT | Prompt Engineering、Model Fine-tuning | NO | Written |
| 12 | (Zhao et al. 2025) | ChatGPT-4 | RAG、Prompt Engineering | NO | Written |
| 13 | (Saad et al. 2025) | ChatGPT-4, Claude-3.0, Gemini-1.5 | Base Model | NO | Written |
| 14 | (Levin, Orkaby, et al. 2025) | ChatGPT-4o, Claude-3.0, Llama 3 8B | Base Model | NO | Written |
| 15 | (Karaçay et al. 2025) | Visual ChatGPT* | Prompt Engineering | NO | Written |
| 16 | (García-Rudolph et al. 2025) | Google Gemini*, ChatGPT-3.5, ChatGPT-4 | Base Model | NO | Written |
| 17 | (Arslan et al. 2025) | ChatGPT*, Copilot* | Base Model | NO | Written |
| 18 | (Shah-Mohammadi and Finkelstein 2025) | ChatGPT-4* | Prompt Engineering | NO | Written |
| 19 | (Ju et al. 2025) | ChatGPT-4 | Undefined | SmartENR AI | Written |
| 20 | (Zhu et al. 2025) | ChatGPT-3.5, ChatGPT-4, ChatGPT-4o, Copilot*, ERNIE Bot-3.5, SPARK*, Qwen-2.5 | Base Model | NO | Written |
| 21 | (Tseng, Huang, and Chen 2025) | ChatGPT*, Copilot* | Base Model | NO | Written |
| 22 | (Soddu et al. 2025) | ChatGPT-4 | Base Model | NO | Written |
| 23 | (Cao et al. 2025) | ERNIE Bot 4.0, Moonshot AI* | Prompt Engineering | NO | Written |
| 24 | (Liu et al. 2025) | ChatGPT-4 | RAG | NO | Written |
| 25 | (Graf, Ritzi, and Schoeler 2025) | Llama3, Phi3 | Prompt Engineering、Model Fine-tuning | NO | Written |
| 26 | (Chen and Liou 2025) | ChatVR-ONCS | Prompt Engineering、Model Fine-tuning | ChatVR-ONCS | Written、Spoken、Visual |
| 27 | (Gilart et al. 2025) | ChatGPT-4 | Prompt Engineering | NO | Written |
| 28 | (Latt et al. 2025) | ChatGPT-3.5, Alice, Azure | RAG、Prompt Engineering | NO | Written |
| 29 | (Kuribara, Hirayama, and Hirata 2025) | ChatGPT-3.5, ChatGPT-4, Copilot* | Base Model | NO | Written |
| 30 | (Ghaffari et al. 2025) | ChatGPT-4 | Base Model | NO | Written |
| 31 | (Wang et al. 2025) | ChatGPT-4o, ChatGPT-4-Turbo | Prompt Engineering | NO | Written |
| 32 | (Niyirora et al. 2025) | TF-IDF text-based vectorization, UMLS semantic mapping, fine-tuned  GPT-4o mini, and Bio-Clinical BERT | Prompt Engineering、Model Fine-tuning | NO | Written |
| 33 | (Lv et al. 2025) | DeepSeek* | Undefined | CLQCS | Written、Spoken、Visual |
| 34 | (Omranian et al. 2025) | LLaMA 3 | Prompt Engineering、Model Fine-tuning | NO | Written |
| 35 | (Wakabayashi and Seto 2025) | ChatGPT-4 | Base Model | NO | Written |
| 36 | (Vithanage et al. 2025) | LLaMA-2-Chat-7B, LLaMA-2-Chat-13B, LLaMA-3-8B, LLaMA3.1–8B, Mistral, Gemini, Qwen, T5-Base, MedAlpaca-7B, MedAlpaca-13B, Baize-Healthcare, ChatDoctor, Asclepius-7B, Asclepius-13B, PMC LLaMA, Me-LLaMA, Clinical-T5-Base | RAG、Prompt Engineering | NO | Written |
| 37 | (Tanrıverdi and Söylemez 2025) | ChatGPT* | Base Model | NO | Written |
| 38 | (Y. Kim, Kim, and Choi 2025) | ChatGPT-4o | Prompt Engineering | NO | Written |
| 39 | (Zhang et al. 2025) | Llama 3.1 8B | RAG、Prompt Engineering | NO | Written |
| 40 | (Yin et al. 2025) | ChatGPT* | Base Model | NO | Written |
| 41 | (Wan et al. 2025) | DeepSeek* | Model Fine-tuning | DeepSeek intelligent data analysis system | Written |
| 42 | (J. Kim, Won, and Lee 2025) | ChatGPT-4 | Base Model | NO | Written |

Note: * indicates that the specific model version was not reported in the original article.
