# Supplementary material for: An Evaluation Framework for Large Language Models in Clinical Nursing: A Scoping Review and Expert Consultation
Source: J Nurs Manag. 2026 Sep 2;2026:9524014. doi: 10.1155/jonm/9524014 (PMC13538949; doi:10.1155/jonm/9524014)
Supplement: Supplementary file 3 [file JONM-2026-9524014-s003.docx]

Appendix 3. Evaluation Framework

| Principle | Dimension | Definition | References |
| --- | --- | --- | --- |
| **Performance and Accuracy** This dimension quantitatively measures the model’s effectiveness on clinical nursing tasks with a definitive “gold standard” or correct answer. It is primarily concerned with objective metrics in classification, prediction, and information retrieval tasks. | Accuracy | In binary or multi-class classification tasks, this metric represents the proportion of correctly predicted instances out of the total number of samples. It is applicable for evaluating tasks such as assessing examination pass rates or diagnosing disease stages. | (Arslan et al. 2025; Cho et al. 2025; García-Rudolph et al. 2025; Graf, Ritzi, and Schoeler 2025; Ju et al. 2025; Karaçay et al. 2025; Y. Kim, Kim, and Choi 2025; J. Kim, Won, and Lee 2025; Kuribara, Hirayama, and Hirata 2025; Latt et al. 2025; Levin, Orkaby, et al. 2025; Levin, Suliman, et al. 2025; Liu et al. 2025; Lv et al. 2025; Omranian et al. 2025; Saad et al. 2025; Shah-Mohammadi and Finkelstein 2025; Soddu et al. 2025; Tanrıverdi and Söylemez 2025; Vithanage et al. 2025; Wan et al. 2024, 2025; Wang et al. 2024, 2025; Wu et al. 2024; Xu et al. 2024; Zhang et al. 2025; Zhao et al. 2025; Zhu et al. 2025) |
|  | Precision/PPV | In classification tasks, this metric quantifies the proportion of true positives among all instances identified as positive by the model. | (Arslan et al. 2025; Cho et al. 2025; Graf et al. 2025; Karaçay et al. 2025; Liu et al. 2025; Niyirora et al. 2025; Omranian et al. 2025; Zaboli et al. 2024; Zhang et al. 2025; Zhu et al. 2025) |
|  | NPV | In classification tasks, this metric quantifies the proportion of true negatives among all instances identified as negative by the model. | (Arslan et al. 2025; Karaçay et al. 2025; Zhu et al. 2025) |
|  | Recall/Sensitivity | In classification tasks, this metric measures the proportion of all actual positive instances that were correctly identified by the model. | (Arslan et al. 2025; Cho et al. 2025; Graf et al. 2025; Karaçay et al. 2025; Liu et al. 2025; Lv et al. 2025; Niyirora et al. 2025; Omranian et al. 2025; Shah-Mohammadi and Finkelstein 2025; Wan et al. 2024; Zaboli et al. 2024; Zhang et al. 2025; Zhu et al. 2025) |
|  | Specificity | In classification tasks, this metric measures the proportion of all actual negative instances that were correctly identified as negative by the model. | (Arslan et al. 2025; Graf et al. 2025; Karaçay et al. 2025; Liu et al. 2025; Shah-Mohammadi and Finkelstein 2025; Wan et al. 2024; Zaboli et al. 2024; Zhu et al. 2025) |
|  | F1 Score | The F1 Score is the harmonic mean of Precision and Recall, providing a single, consolidated metric that reflects a model's overall performance. | (Cho et al. 2025; Liu et al. 2025; Lv et al. 2025; Niyirora et al. 2025; Omranian et al. 2025; Scroggins et al. 2025; Shah-Mohammadi and Finkelstein 2025; Vithanage et al. 2025; Zhang et al. 2025; Zhu et al. 2025) |
|  | F2 Score | The F2 Score is a weighted harmonic mean of Precision and Recall that places twice as much emphasis on Recall as on Precision. | (Cho et al. 2025) |
|  | BERT Score | BERT Score is an automated evaluation metric that quantifies the semantic similarity between a candidate text (model-generated output) and a reference text (gold standard) by leveraging contextual word embeddings from the BERT model. | (Vithanage et al. 2025; Zhang et al. 2025) |
|  | AUC | AUC represents the area under the ROC curve, providing a single, aggregate measure of a model's overall ability to discriminate between positive and negative classes across all possible classification thresholds. | (Arslan et al. 2025; Karaçay et al. 2025; Wan et al. 2025; Zaboli et al. 2024) |
| **Clinical Validity and Safety** This dimension assesses the clinical appropriateness, quality, and potential for harm of the model's generated content. This evaluation is typically qualitative and requires subjective assessment by clinical experts to ensure the output is sound, safe, and contextually relevant to patient care. | Factuality | Factuality evaluates whether the model’s generated content is free from fabricated or erroneous clinical information that could mislead clinical decision-making or result in patient harm. | (Ghaffari et al. 2025; Y. Kim et al. 2025; J. Kim et al. 2025; Latt et al. 2025; Soddu et al. 2025; Wan et al. 2024; Zhao et al. 2025) |
|  | Consistency | Consistency assesses the degree to which the model-generated content aligns with established clinical guidelines, evidence-based best practices, and the consensus of clinical experts. | (Arslan et al. 2025; Cao et al. 2025; Ghaffari et al. 2025; Gosak et al. 2025; Johnson et al. 2025; Karaçay et al. 2025; J. Kim et al. 2025; Latt et al. 2025; Levin, Orkaby, et al. 2025; Niyirora et al. 2025; Tanrıverdi and Söylemez 2025; Tseng, Huang, and Chen 2025; Wan et al. 2024; Wang et al. 2025; Woodnutt et al. 2024; Zaboli et al. 2024; Zhang et al. 2025) |
|  | Completeness | Completeness evaluates the completeness of the model-generated content, assessing whether it covers all critical and necessary information points without significant omissions. | (Gilart et al. 2025; Gosak et al. 2025; Ju et al. 2025; Y. Kim et al. 2025; Latt et al. 2025; Soddu et al. 2025; Wan et al. 2024) |
|  | Relevance | Relevance evaluates how closely the model-generated content aligns with the specific clinical scenario and informational needs embedded in the user's prompt. | (Gilart et al. 2025; Gosak et al. 2025; Y. Kim et al. 2025; J. Kim et al. 2025; Saad et al. 2025; Scroggins et al. 2025) |
|  | Clinical Prioritization | Clinical Prioritization evaluates the model's ability to correctly rank or sequence nursing interventions and care plan components according to their clinical importance and urgency. | (Cao et al. 2025; Johnson et al. 2025) |
|  | Error Type | Error Type focuses on identifying, categorizing, and understanding the patterns of errors present in the model's generated content. | (Cao et al. 2025; Cho et al. 2025; García-Rudolph et al. 2025; Ghaffari et al. 2025; Y. Kim et al. 2025; Kuribara et al. 2025; Liu et al. 2025; Lv et al. 2025; Omranian et al. 2025) |
|  | Guidance | Guidance assesses whether the model’s output provides clear, clinically appropriate, and safe recommendations for subsequent steps. | (Latt et al. 2025) |
| **Workflow Integration and Efficiency** This dimension evaluates the model's practical impact on existing clinical nursing workflows. It measures the degree to which the LLM streamlines processes, reduces administrative burden, and enhances overall productivity in a real-world setting. | Response Time | Response Time measures the time elapsed from the moment a user submits a query to the LLM to the moment the model delivers a complete response. | (Ghaffari et al. 2025; Gilart et al. 2025; Ju et al. 2025; J. Kim et al. 2025; Levin, Orkaby, et al. 2025; Levin, Suliman, et al. 2025; Saad et al. 2025; Wan et al. 2025) |
|  | Task Completion Efficiency | Task Completion Efficiency assesses the model's direct impact on nursing productivity by measuring changes in the time and effort required to complete clinical tasks. It is evaluated through both objective and subjective methods. | (Ju et al. 2025; Lv et al. 2025; Wakabayashi and Seto 2025; Yin et al. 2025) |
|  | Communication Efficiency | Communication Efficiency measures the average number of conversational turns—a single user prompt followed by a single model response-required to fully address a user's initial clinical query or complete a given task. | (Wan et al. 2024) |
|  | Conciseness | Conciseness quantitatively assesses the length of the model's generated output, typically measured by word or character count. It is used to evaluate whether the response is appropriately brief and to the point or unnecessarily verbose for the given task. | (Latt et al. 2025; Levin, Suliman, et al. 2025; Saad et al. 2025; Zhao et al. 2025) |
| **Usability and User Experience** This dimension evaluates the subjective experience of clinical end-users when interacting with the LLM. | Satisfaction | Satisfaction provides a summative measure of the end-user's overall subjective feeling and contentment with their interactions with the LLM. | (J. Kim et al. 2025; Lv et al. 2025; Wan et al. 2024; Xu et al. 2024; Yin et al. 2025) |
|  | Perceived Usefulness | Perceived Usefulness measures the user's subjective belief that using the LLM will enhance their job performance and effectiveness. | (Chen and Liou 2025; Ju et al. 2025; J. Kim et al. 2025) |
|  | Ease of Use | Ease of Use measures the degree to which a user believes that interacting with the LLM will be free of physical and, more importantly, mental effort. | (Chen and Liou 2025; Ju et al. 2025; J. Kim et al. 2025) |
|  | Affective Interaction Quality | Affective Interaction Quality assesses the emotional quality of the interaction by evaluating both the degree of empathy and emotional support conveyed in the model's responses and the frequency of negative emotions, such as frustration, exhibited by the user. | (Wan et al. 2024; Wang et al. 2025; Xu et al. 2024) |
|  | Fluency | Fluency assesses whether the output is grammatically correct, logically coherent, and phrased in a manner that is natural and fluent | (Gilart et al. 2025; Ju et al. 2025; J. Kim et al. 2025; Latt et al. 2025) |
| **Model Reliability and Ethical Considerations**  This dimension assesses the fundamental soundness and dependability of the LLM for clinical applications. | Output Stability | Output Stability evaluates the consistency of the quality and content of the model's output when presented with semantically similar prompts or slight variations of the same input. | (Cho et al. 2025; Wang et al. 2024, 2025; Wu et al. 2024) |
|  | Robustness | Robustness evaluates the model’s ability to maintain its performance and functional correctness when confronted with imperfect inputs, such as typographical errors, common clinical abbreviations, grammatical variations, or intentionally adversarial perturbations. | (Vithanage et al. 2025) |
|  | Fairness/Bias | Fairness/Bias assesses whether the model's outputs exhibit systematic, inequitable, or prejudicial variations when applied to different patient populations | (Vithanage et al. 2025) |
|  | Explainability | Explainability evaluates the model's capacity to provide a clear, clinically sensible, and understandable rationale or evidence for its outputs, such as its recommendations, classifications, or conclusions. | (Wan et al. 2024) |
|  | speculative vocabulary | speculative vocabulary provides a systematic assessment of the model's use of speculative, hedging, or uncertain language. | (Zhao et al. 2025) |
|  | Seniority | Seniority is a critical contextual attribute, not a direct performance measure, that documents the time elapsed from a model's knowledge cutoff date or its official release date to the time the study was conducted. | (Levin, Orkaby, et al. 2025) |
|  | Consistency of Accuracy Across Days | Consistency of Accuracy Across Days evaluates the temporal stability of a model's performance by assessing whether its accuracy and other key performance indicators remain consistent across different days or distinct phases of a longitudinal study period. | (Arslan et al. 2025; Vithanage et al. 2025) |
| **Competency Development**  This dimension evaluates the perceived impact of the LLMs on the professional growth of its clinical users. It focuses on the enhancement of their clinical knowledge, skills, and overall professional competence. This is a user-centric measure that assesses the educational and developmental value of the tool, rather than its intrinsic technical performance. | AI Literacy | This metric quantifies the extent to which interacting with the LLM-based tool improves a clinical user's AI literacy | (Tseng et al. 2025) |
|  | Usage Rate | This metric quantitatively measures how often and consistently clinical users engage with the LLM-based tool or platform | (Tseng et al. 2025) |
|  | Enhancement of Communication Skills | This metric assesses the user's perceived improvement in their communication capabilities and techniques as a direct result of interacting with and utilizing the LLM-based tool. | (Chen and Liou 2025; J. Kim et al. 2025) |
|  | Critical Thinking | This metric evaluates the influence of the LLM-based tool on a nurse's ability to engage in critical thinking and complex clinical reasoning. | (Yin et al. 2025) |
|  | Enhancement of Clinical Knowledge and Skills | This metric assesses the user's perceived improvement in their clinical knowledge base and the practical application of nursing skills resulting from the use of the LLM-based tool. | (Wan et al. 2025; Yin et al. 2025) |
