# Supplementary material for: An Evaluation Framework for Large Language Models in Clinical Nursing: A Scoping Review and Expert Consultation
Source: J Nurs Manag. 2026 Sep 2;2026:9524014. doi: 10.1155/jonm/9524014 (PMC13538949; doi:10.1155/jonm/9524014)
Supplement: Supplementary file 4 [file JONM-2026-9524014-s004.docx]

Appendix 4. Evaluation Scenarios for Large Language Models

| Level 1 evaluation scenario | Level 2 evaluation scenario | References |
| --- | --- | --- |
| Clinical decision support and knowledge retrieval | Nursing care plan formulation | Woodnutt et al., 2024, Gosak et al., 2025, Johnson et al., 2025, Cao et al., 2025, Gilart et al., 2025 |
|  | Assisting nursing interventions | Wang et al., 2024, Xu et al., 2024, Levin et al., 2025a, Zaboli et al., 2024, Levin et al., 2025b, Karaçay et al., 2025, Saad et al., 2025, Ju et al., 2025 |
|  | Triage | Wan et al., 2024, Arslan et al., 2025, Liu et al., 2025, Wang et al., 2025 |
|  | Assisting diagnosis | Soddu et al., 2025 |
| Clinical nursing education | Nursing knowledge testing | Wu et al., 2024, Zhao et al., 2025, García-Rudolph et al., 2025, Zhu et al., 2025, Kuribara et al., 2025 |
|  | Clinical scenario simulation | Chen and Liou, 2025, Ghaffari et al., 2025, Yin et al., 2025 |
|  | Optimization of clinical nursing education | Tseng et al., 2025, Tanrıverdi and Söylemez, 2025, Kim et al., 2025a |
| Patient communication and health education | Provide sexual health information | Latt et al., 2025 |
|  | Identifying health problems | Scroggins et al., 2025 |
| Nursing quality Management | Adverse event reporting | Cho et al., 2025 |
|  | Staff well-being and burnout assessment | Omranian et al., 2025 |
|  | Process management in clinical nursing | Wan et al., 2025 |
| clinical documentation and information management | Identification from nursing reports | Shah-Mohammadi and Finkelstein, 2025, Graf et al., 2025 |
|  | Nursing documentation or record organization | Niyirora et al., 2025, Lv et al., 2025, Wakabayashi and Seto, 2025, Vithanage et al., 2025, Kim et al., 2025b, Zhang et al., 2025 |
