# Supplementary material for: An Evaluation Framework for Large Language Models in Clinical Nursing: A Scoping Review and Expert Consultation
Source: J Nurs Manag. 2026 Sep 2;2026:9524014. doi: 10.1155/jonm/9524014 (PMC13538949; doi:10.1155/jonm/9524014)
Supplement: Supplementary file 5 [file JONM-2026-9524014-s005.docx]

### Appendix 5： Search Query

**Database: PubMed**

("large language models"[MeSH] OR "large language model"[Title/Abstract] OR "generative artificial intelligence"[Title/Abstract] OR "LLM"[Title/Abstract] OR "LLMs"[Title/Abstract] OR "generative pre trained transformer"[Title/Abstract] OR "chatgpt*"[Title/Abstract] OR "chatgpt"[Title/Abstract] OR "GPT"[Title/Abstract] OR "GPT"[Title/Abstract] OR "Bard"[Title/Abstract] OR "Bing-Chat"[Title/Abstract] OR "LaMDA"[Title/Abstract] OR "LLaMA"[Title/Abstract] OR "Gemini"[Title/Abstract] OR "Claude"[Title/Abstract] OR "hugging face"[Title/Abstract] OR "DeepSeek"[Title/Abstract] OR "PaLM"[Title/Abstract] OR "Transformer"[Title/Abstract]) AND ("Nursing"[MeSH] OR "Nurs*"[Title/Abstract] OR "clinical nurs*"[Title/Abstract] OR "Nursing Care Plan"[Title/Abstract] OR "Patient Care"[Title/Abstract] OR "Patient Care Planning"[Title/Abstract] )

Results: 1135

**Database: Web Of Science**

(TS=(“Large Language Models” OR “large language model” OR “generative artificial intelligence” OR LLM OR LLMs OR “Generative Pre-trained Transformer” OR ChatGPT* OR chatgpt OR GPT OR gpt OR Bard OR Bing-Chat OR LaMDA OR LLaMA OR Gemini OR Claude OR “Hugging Face” OR DeepSeek OR PaLM OR Transformer)) AND (TS=(Nursing OR Nurs* OR“clinical nurs*”OR“Nursing Care Plan”OR “Patient Care”OR“Patient Care Planning”))

Results: 3113

**Database: Cochrane Library**

((large language models):ab,ti,kw OR (large language model):ab,ti,kw OR (generative artificial intelligence):ab,ti,kw OR (LLM):ab,ti,kw OR (LLMs):ab,ti,kw OR (Generative Pre-trained Transformer):ab,ti,kw OR (ChatGPT*):ab,ti,kw OR (chatgpt):ab,ti,kw OR (GPT):ab,ti,kw OR (gpt):ab,ti,kw OR (Bard):ab,ti,kw OR (Bing-Chat):ab,ti,kw OR (LaMDA):ab,ti,kw OR (LLaMA):ab,ti,kw OR (Gemini):ab,ti,kw OR (Claude):ab,ti,kw OR (Hugging Face):ab,ti,kw OR (DeepSeek):ab,ti,kw OR (PaLM):ab,ti,kw OR (Transformer):ab,ti,kw) AND ((Nurs*):ab,ti,kw OR (clinical nurs* ):ab,ti,kw OR (Nursing Care Plan):ab,ti,kw OR (Patient Care):ab,ti,kw OR

(Patient Care Planning):ab,ti,kw )

Results: 494

**Database: Embase**

('large language model'/exp OR 'large language model':ab,ti OR 'generative artificial intelligence':ab,ti OR'llm':ab,ti OR 'llms':ab,ti OR 'generative pre-trained transformer':ab,ti OR 'chatgpt*':ab,ti OR 'chatgpt':ab,ti OR 'gpt':ab,ti OR 'bard':ab,ti OR 'bing-chat':ab,ti OR 'lamda':ab,ti OR 'llama':ab,ti OR 'gemini':ab,ti OR 'claude':ab,ti OR 'hugging face':ab,ti OR 'deepseek':ab,ti OR 'palm':ab,ti OR 'transformer':ab,ti) AND ('Nursing'/exp OR 'Nurs* ':ab,ti OR 'clinical nurs*':ab,ti 'Nursing Care Plan':ab,ti OR'Patient Care ':ab,ti OR 'Patient Care Planning':ab,ti )

Results: 843
