# Supplementary material for: An Evaluation Framework for Large Language Models in Clinical Nursing: A Scoping Review and Expert Consultation
Source: J Nurs Manag. 2026 Sep 2;2026:9524014. doi: 10.1155/jonm/9524014 (PMC13538949; doi:10.1155/jonm/9524014)
Supplement: Supplementary file 6 [file JONM-2026-9524014-s006.docx]

**Appendix 6. Proposed minimum technical reporting checklist for studies evaluating LLM in clinical nursing practice**

**Note:** This checklist is a preliminary synthesis of existing reporting recommendations adapted for studies evaluating LLMs in clinical nursing tasks. It represents the authors’ recommended minimum reporting checklist and is intended to support more complete, transparent, and reproducible reporting. It should not be regarded as a formally developed reporting guideline. “N/A” indicates not applicable.

| **Item** | **Reporting domain** | **Information to be reported** | **Examples or notes** | **Yes/No/NA** | **PAGE** |
| --- | --- | --- | --- | --- | --- |
| 1 | Model identification | Model name and version | GPT-4o or GPT-4o-2024-08-06 |  |  |
|  |  | Developer/provider | Report the organization that developed or provides the model. |  |  |
| 2 | Access and deployment | Model access method | Report how the model was accessed, such as through a public web interface, mobile application, API, or locally developed software interface. |  |  |
|  |  | Deployment environment | Report where and how the model was deployed, such as in a public cloud, private cloud, local or on-premises environment, or institution-hosted private environment. |  |  |
| 3 | Prompt and generation settings | Generation parameters | Report configurable generation parameters, such as temperature, top-p, maximum output length, random seed, and stop sequences. |  |  |
|  |  | Prompt design and refinement process | Report the prompt structure and any pilot testing or iterative refinement. Provide the complete prompts where possible. |  |  |
|  |  | Prompt execution setup | Report the conversation format, number of repeated runs, and method for handling multiple outputs. |  |  |
| 4 | Clinical nursing task and input data characteristics | Clinical nursing task. | Report the specific clinical nursing task performed by the model, such as nursing assessment. |  |  |
|  |  | Input data modality, source | Modalities may include text, images, structured numerical data, or multimodal data; sources may include electronic health records, medication orders, or handover records. |  |  |
| 5 | Model adaptation and knowledge integration | Model adaptation, augmentation, and constraint strategies | Report any model adaptation, augmentation, or constraint strategies used, such as retrieval-augmented generation, fine-tuning, rule-based constraints, or structured templates. State if the unmodified model was evaluated. |  |  |
|  |  | External knowledge sources | Where applicable, report the external knowledge sources used to support model generation, such as clinical guidelines, nursing standards, hospital policies, or local databases. |  |  |
| 6 | Output generation and processing | Output format and structure | Report the expected and generated format and level of structure of the model outputs |  |  |
|  |  | Output post-processing and handling | Report any procedures used to select, aggregate, edit, transform, or code model outputs. |  |  |
| 7 | Reproducibility conditions | Testing conditions and material availability | Report the testing date, model version or update status, and the availability of prompts, code, and evaluation materials, where applicable. |  |  |

*Abbreviations: LLM = large language model; API = application programming interface; RAG = retrieval-augmented generation; NA = not applicable.*
